# Short linear motifs – *ex nihilo* evolution of protein regulation

**DOI:** 10.1186/s12964-015-0120-z

**Published:** 2015-11-21

**Authors:** Norman E. Davey, Martha S. Cyert, Alan M. Moses

**Affiliations:** Conway Institute of Biomolecular and Biomedical Sciences, University College Dublin, Dublin 4, Ireland; Department of Biology, Stanford University, Stanford, CA 94305 USA; Department of Cell & Systems Biology, University of Toronto, Toronto, Canada; Centre for the Analysis of Genome Evolution and Function, University of Toronto, Toronto, Canada

**Keywords:** Motifs, Short linear motifs, SLiMs, Cis-regulatory elements, RNA motifs, Evolution, Modularity, Protein evolution

## Abstract

Short sequence motifs are ubiquitous across the three major types of biomolecules: hundreds of classes and thousands of instances of DNA regulatory elements, RNA motifs and protein short linear motifs (SLiMs) have been characterised. The increase in complexity of transcriptional, post-transcriptional and post-translational regulation in higher Eukaryotes has coincided with a significant expansion of motif use. But how did the eukaryotic cell acquire such a vast repertoire of motifs? In this review, we curate the available literature on protein motif evolution and discuss the evidence that suggests SLiMs can be acquired by mutations, insertions and deletions in disordered regions. We propose a mechanism of *ex nihilo* SLiM evolution – the evolution of a novel SLiM from “nothing” – adding a functional module to a previously non-functional region of protein sequence. In our model, hundreds of motif-binding domains in higher eukaryotic proteins connect simple motif specificities with useful functions to create a large functional motif space. Accessible peptides that match the specificity of these motif-binding domains are continuously created and destroyed by mutations in rapidly evolving disordered regions, creating a dynamic supply of new interactions that may have advantageous phenotypic novelty. This provides a reservoir of diversity to modify existing interaction networks. Evolutionary pressures will act on these motifs to retain beneficial instances. However, most will be lost on an evolutionary timescale as negative selection and genetic drift act on deleterious and neutral motifs respectively. In light of the parallels between the presented model and the evolution of motifs in the regulatory segments of genes and (pre-)mRNAs, we suggest our understanding of regulatory networks would benefit from the creation of a shared model describing the evolution of transcriptional, post-transcriptional and post-translational regulation.

## Background

Over the past 20 years our understanding of genome organisation expanded rapidly as researchers leveraged breakthroughs in sequencing technology to determine the complete DNA sequence of numerous eukaryotic genomes. It quickly became clear that these genomes differed in several important ways from the prokaryotic genomes that preceded them. Perhaps the most obvious difference was that eukaryotic genomes contained a much larger proportion of non-coding DNA than their distant prokaryotic relatives. In the first decade of the 21^st^ century, the genomics community turned to identifying the complete repertoire of functional elements in these non-coding regions. This led to a flurry of research to understand the function and evolution of the human genome’s vast “heart of darkness” [1], culminating with ENCODE and related projects [2–4]. Over the same period of time surprising discoveries were causing a similar transition in thinking about the protein products of the eukaryotic genomes [5, 6]. Structural studies were revealing that a substantial number of proteins or segments of proteins in complex organisms are intrinsically disordered, lacking a stable well-defined tertiary structure in their native state [7, 8]. Moreover, these regions were shown to perform numerous functions - directly contradicting the structure-function paradigm, a basic tenet of structural biology [6, 9–11]. These observations, like the analogous discovery of the extensive functionality of non-coding regions, forced a paradigm shift and sparked an interest in these hitherto underappreciated regions.

Many of the interactions mediated by these regions were observed to be low-affinity. Consequently, they often mediate interactions where the biological requirements are such that a transient or dynamic binding event is preferable [10, 12]. Unexpectedly, the vast majority of these modules were shown to be encoded in short regions, what we now describe as short linear motifs (or SLiMs), of less than ten amino acids that mediate transient interactions with peptide binding domains [13]. Furthermore, within these peptides, as few as three or four residues typically encoded the majority of affinity and specificity of binding [10, 14]. Despite these barriers to motif discovery the census of modules rapidly expanded and thousands of SLiMs have now been functionally characterised [9]. They are known to be involved in a diverse array of functions: they assist in protein complex assembly; recruit substrates to modifying enzymes; control protein stability; direct trafficking to and anchoring in specific subcellular locations; and act as sites of post-translational modification (PTM) moiety addition or removal, proteolytic cleavage and structural modification [9, 10, 12, 13]. However, despite increasing appreciation of their abundance and importance [10, 15], little was known until recently about SLiM evolution: especially in comparison to globular domain evolution whose duplication, divergence and recombination was already textbook knowledge [16, 17]. Nevertheless, consideration of the potential evolutionary plasticity of the compact and degenerate SLiMs led to the hypothesis that they could play key roles in protein evolution [16]: acquiring a novel SLiM is an appealing mechanism whereby a protein can gain important regulatory functions. Therefore protein networks could acquire new interactions with only a few amino acid changes [16]. Indeed, short DNA regulatory motifs were thought to be key substrates for transcriptional regulatory evolution [18], and a parallel with protein motifs seemed possible [16].

In the past 10 years, there has been much progress in testing the hypothesis that the gain and loss of SLiMs can underlie evolutionary changes in protein function. Here, we review illustrative examples of SLiM evolution and large-scale efforts to characterise the evolutionary diversity of SLiMs. In doing so, we identify several outstanding questions about the origin and evolution of SLiMs: What are the evolutionary forces that drive motif evolution? What is the mechanism of motif binding pocket evolution? When did extensive motif use evolve? Finally, we discuss the parallels in motif evolution at the transcriptional, post-transcriptional and post-translational regulation level.

## The evolutionary properties of short linear motifs

**Fig. 1:**
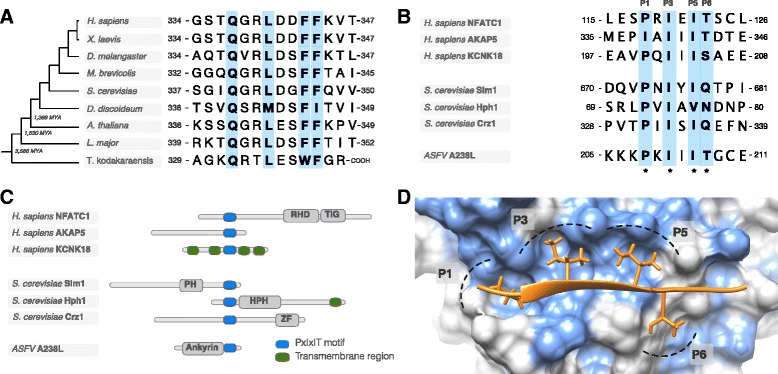
Conservation of functionally important motifs and the proliferation of motifs through *ex nihilo* motif acquisition. **a** Alignment of the PCNA-binding PIP box motif of Flap endonuclease 1 (FEN1) showing the motif conservation spanning over 3 billion years of evolution across all Eukaryotes and Archaea (representative species - *Thermococcus kodakaraensis*) [24, 25, 108]. **b** An alignment of a representative selection of PxIxIT motif instances: Nuclear factor of activated T-cells, cytoplasmic 1 (NFATC1) [109], A-kinase anchor protein 5 (AKAP5) [110] and Potassium channel subfamily K member 18 (KCNK18) [111] from human; Phosphatidylinositol 4,5-bisphosphate-binding protein SLM1 (Slm1) [112], Protein HPH1 (Hph1) [113] and Transcriptional regulator CRZ1 (Crz1) from yeast [114]; and Ankyrin repeat domain-containing protein A238L from African swine fever virus (ASFV) [115]. Each motif instance occurs in a non-homologous protein (see panel **c**) and the most likely mode of acquisition for these functional modules is by *ex nihilo* evolution through random mutation. The alignment shows a clear preference for specific residues at a given position in the peptide with each position allowing a different level of degeneracy. These preferences reflect the preferences of the Calcineurin PxIxIT binding pocket (see panel **d**). **c** The modular architecture of the proteins from panel B showing the distinct organisation of the non-homologous proteins. Domains (grey), transmembrane regions (green) and PxIxITs (blue) are shown. Proteins are aligned around the PxIxIT instances. **d** Structure of the PxIxIT binding pocket of the human calcineurin catalytic A subunit bound to the PxIxIT of African swine fever virus A238L (PDB ID:4F0Z) [115]. The peptide binds by beta-augmentation and the defined residues at P1, P3, P5 sit in a conserved hydrophobic pocket explaining the strong preferences at these positions in known PxIxIT instances (light blue surface on the domain denotes hydrophobic residues) [109, 110, 116]

**Fig. 2:**
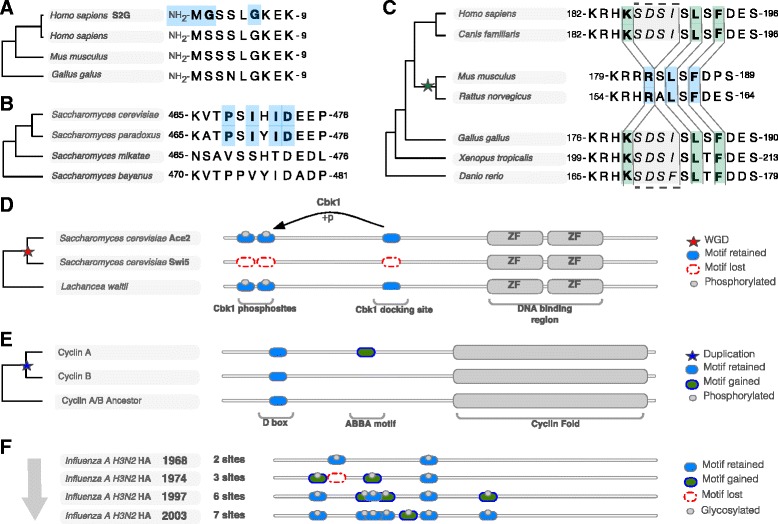
Examples of *ex nihilo* motif gain and motif loss. **a** The N-terminus of the SHOC2 contains an *S2- > G* mutation in multiple Noonan-like syndrome patients that “knocks in” an N-myristoylation motif [26]. *Blue bold* residues signify the specificity determining residues of the motif. **b** A PxIxIT calcineurin-docking motif in *S. cerevisiae* Serine/threonine-protein kinase ELM1 (Elm1) has likely evolved in the common ancestor of *S. cerevisiae* and *S. paradoxus* [27]. **c** A human-centric phylogeny of E3 ubiquitin-protein ligase Mdm2 (Mdm2). An RxL Cyclin docking motif was gained in the rodent Mdm2 proteins as a result of a four amino acid deletion (*grey region*) [117]. *Green bold* residues signify the position of the residues corresponding to the specificity determining residues of the motif before the SDSI deletion. **d** Example of motif loss contributing to functional divergence post-duplication. *S. cerevisiae* ohnologues Ace2 and Swi5 were both retained after the whole genome duplication (WGD) but have functionally diverged post duplication, in part, by the loss of a serine/threonine-protein kinase Cbk1 docking site and two Cbk1 phosphosites in the Swi5 lineage. A representative example of a single pre-WGD homologue in *Lachancea waltii* shows the modular architecture of the Ace2/Swi5 ancestor [36]. **e** Example of motif gain contributing to functional divergence post-duplication. The Cyclin A and Cyclin B regulatory subunits of the CDK family protein kinases share a common ancestor that contained a D box motif to recruit the APC/C E3 ubiquitin ligase promoting Cyclin destruction during mitosis. Post-duplication the Cyclin A lineage gained an ABBA motif allowing Cyclin A to be destroyed earlier than Cyclin B during prometaphase [40]. **f** The accumulation of the Nx[TS] glycosylation motifs in hemagglutinin of *Influenza* H3N2 over the last 40 years. The number of glycosylation motifs has increased from two to seven tuning the trade-off between host receptor binding and immune evasion [118]

Historically, SLiMs were discovered as islands of conservation in rapidly evolving regions and, as a result, many of the early motif instances were conserved over large taxonomic ranges [19–22]. Consequently, it has long been clear that a substantial number of motifs with important functions are under strong purifying selection against deleterious mutations [23]. For example, the PCNA-binding PIP box motif in Flap endonuclease 1 (FEN1) is conserved across all Eukaryotes [24] and Archaea [25] surviving over three billion years of evolution (Fig. 1a). Furthermore, SLiMs recognised by the same motif-binding pocket are typically found in multiple non-homologous proteins (Fig. 1b-d). This led to the proposal of a mechanism of motif acquisition driven by *ex nihilo* motif birth by random mutation [16]. However, motif birth had not been directly observed. This posed a fundamental question about motif evolution – how common is *ex nihilo* motifs motif birth from random sequence? A pioneering study of patients with Noonan-like syndrome revealed that several patients have *de novo S2- > G* substitutions in human leucine-rich repeat protein SHOC-2 (SHOC2) that result in the *ex nihilo* birth of a myristoylation motif [26] (Fig. 2a). Remarkably, this mutation was shown to have occurred independently on multiple occasions and for all individuals where the parental sequence was tested the substitution was absent in the parents. These observations suggested that random mutation can drive *ex nihilo* motif birth and that alleles with novel motifs may be common in a population [26].

Over the past decade, several analyses tracing the taxonomic range of motifs have shown that SLiMs are regularly gained and lost by individual lineages (see Table 1, Fig. 2a-f). A recent unbiased proteome-wide analysis of the calcineurin (Ca2+/calmodulin-dependent phosphatase) binding PxIxIT docking motif in *Saccharomyces cerevisiae* revealed that approximately 70 % of PxIxIT sites are limited to the *Saccharomyces sensu stricto* clade and therefore have evolved within the past 20 million years [27] (Fig. 2b). The extensive datasets provided by high-throughput proteomic studies corroborate these observations by repeatedly returning a large number of motifs that are clade specific [28, 29] and by revealing that SLiM-mediated interactions are rapidly rewired compared to other classes of protein-protein interaction [30–33]. Interestingly, despite the evolutionary transience of individual motif instances, interaction networks are often conserved. Many yeast Cyclin-dependent kinase 1 (Cdk1) phosphorylation motifs are evolutionarily transient but the presence of a modification site(s) in a given protein region is conserved [29]. Similarly, the acidophilic caspase family cleavage site motifs are often lost in orthologous proteins, however, are gained in different members of a targeted pathway thereby conserving network functionality [34]. This process of motifs appearing and disappearing while preserving the same interactions is sometimes referred to as “turnover” [35]. The development of distinct protein functionality either post-duplication or after *de novo* gene birth also provides insights into motif gain and loss [36] (see Table 1). Gene duplication often results in alteration of the transcriptional, post-transcriptional or post-translational control of the paralogues [37]. Many paralogous proteins acquire distinct functionality by gaining or losing SLiMs [38, 39] that result in differential regulation [36, 40] or subfunctionalisation [41, 42] (Fig. 2d-e). *De novo* gene birth, the gain of a novel transcribed and translated gene, has recently been revealed to be relatively common [43]. Currently, few proteins resulting from recent *de novo* gene birth have been functionally characterised, and examples of motif-containing novel proteins are even rarer. However, instances from HIV accessory proteins, considered to be products of *de novo* gene birth, suggest that motif acquisition may be a common route for a novel protein to gain functional modules [44–46] (see Table 1).

**Table 1:** Table of characterised examples of motif gain and loss modulating protein function

Species protein (Gene)	Motif	Sequence^a^	Evolution	Function
Ex nihilo motif acquisition				
*H. sapiens* Leucine-rich repeat protein SHOC-2 (SHOC2)	N-myristoylation motif	_1_ **MG**SSL**G**_6_	Allele with a single *S2- > G* mutation in SHOC2 in Noonan-like syndrome patients “knocks in” a motif [26].	N-myristoylation of SHOC2
*H. sapiens* Adapter molecule crk (CRK)	ABL1 SH3 domain binding motif	_69_PPV**P**PS**P**AQP_78_	*Ex nihilo* acquisition in early mammals [85].	Recruitment of ABL1 to CRK
*S.cerevisiae* Cell division control protein 6 (Cdc6)	SCF Cdc4 degrons	_364_QVP**LTP**TT**S**PVK_375_	*Ex nihilo* acquisition in *Saccharomyces sensu stricto* clade [57].	Degradation of Cdc6 by the SCF E3 Ub ligase
*M. musculus* E3 ubiquitin-protein ligase Mdm2 (Mdm2)	Cyclin binding motif	_179_KKRR**R**S**L**S**F**DPS_189_	Acquisition in the rodent lineage via a four amino acid deletion [117].	Recruitment of and phosphorylation by CDK2
*D. melanogaster* Segmentation polarity homeobox protein engrailed (en)	Groucho interacting motif	_198_QAS**IFRPF**EAS_208_	*Ex nihilo* acquisition in *diptera*/*lepidoptera* [134].	Recruitment of groucho
*S.cerevisiae* Serine/threonine-protein kinase ELM1 (Elm1)	Calcineurin docking motif	_465_KVT**P**S**I**H**ID**EEP_476_	*Ex nihilo* acquisition in *Saccharomyces* sensu stricto clade [27].	Recruitment of and dephosphorylation by calcineurin
*Influenza* hemagglutinin (H3)	N-glycosylation motifs	Five NxT sites	Strains spanning the last 40 years have shown gradual acquisition of five novel N-glycosylation sites [118].	Increased immune system evasion and decreased infectivity
Motif gain/loss post duplication				
*S. cerevisiae* Spindle assembly checkpoint component MAD3 (Mad3)	APC/C Cdc20 binding KEN box	_27_ETQ**KEN**ILP_35_	Lost in the Bub1 functional homologues after Mad3-like/Bub1-like duplications [41].	Loss of APC/C inhibitory function
*S. cerevisiae* Metallothionein expression activator (Ace2)	Cbk1 docking motif	_280_NGG**Y**Q**FPP**PTL_291_	Lost in Swi5 after Swi5/Ace2 duplication [36].	Loss of Cbk1 regulated localisation
*H. sapiens* Cyclin-A2 (CCNA2)	APC/C CDC20 binding ABBA motif	_96_QPA**F**T**IH**V**D**EAE_108_	*Ex nihilo* acquisition in Cyclin A after the Cyclin A/Cyclin B duplication [40].	Early degradation during an active spindle assembly checkpoint
Tuning of motif specificity/affinity				
*S. cerevisiae* MAP kinase kinase Pbs2 (Pbs2)	Sho1 SH3 domain binding motif	_90_IVN**K**PL**P**PL**P**VAG_102_	Only binds the SH3 domain of yeast Sho1 but can be recognised by multiple non-yeast SH3 domains [53].	Specific interaction with Sho1
*S. cerevisiae* Peroxisomal membrane protein PEX14 (Pex14)	Pex14 SH3 domain binding motif	_84_AMP**P**TL**P**H**R**D_93_	Promiscuous *in vitro* but only binds the SH3 domain of co-localised Pex13 *in vivo* [53].	Promiscuous in vitro interactions
Ex nihilo co-operative/competitive interface evolution				
*S. cerevisiae* N-acetyltransferase ECO1 (Eco1)	SCF Cdc4 degron	_90_TGT**ITP**LN**S**SPL_101_	*Ex nihilo* acquisition in the *Saccharomycetaceae* clade of four motifs required for sequential kinase and ubiquitin ligase recruitment [58].	Degradation of Eco1 by the SCF E3 Ub ligase
	Mck1 modification site	_91_GTI**T**PLN**S**SPL_101_		
	Cdc7 modification site	_95_PLN**SS**PLKK_103_		
	Cdk1 modification site	_96_LNS**SP**LK**K**SS_105_		
*S. cerevisiae* DNA replication licensing factor MCM3 (Mcm3)	Cdk1 modification sites	_758_SKR**SP**Q**K**SPK_767_	*Ex nihilo* acquisition of a cluster of Cdk1 modification sites in the *Saccharomycetaceae* family [54].	Regulation of nucleocytoplasmic shuttling of Mcm3
		_762_PQK**SP**K**K**RQR_771_		
*S. cerevisiae* DIG2 (Dig2)	MAPK D-site	_97_HSL**KRKR**VPPA**L**N**F**SDI_113_	Acquisition of MAPK D-site followed by *ex nihilo* acquisition of overlapping calcineurin-binding PxIxIT site; Dig1 paralog contains D-site but no PxIxIT [27, 135].	Competitive recognition of substrate by kinase and phosphatase
	Calcineurin PxIxIT	_103_RVP**P**A**L**N**FS**IQA_114_		
*H. sapiens* Retinoblastoma-associated protein RB (RB1)	Cyclin A docking site	_873_ **K**K**L**R**F**_875_	Co-evolution of PP1 and Cyclin A recognition motifs [56, 136].	Competitive recognition of substrate by kinase and phosphatase
	PP1 binding RVxF	_873_K**KL**R**F**_877_		
Motif gain/loss post de novo gene birth				
Human immunodeficiency virus type 1 (HIV-1) Protein Vpu (vpu)	SCF β-TrCP degron	_48_RAE**DSG**NE**S**EGE_59_	*Ex nihilo* acquisition in the novel overprinted Vpu protein, present only HIV-1 and its simian precursors [44, 45].	Highjacking of the host SCF-β-TrCP E3 Ub ligase

^a^Sequence overlapping motif - the major specificity and affinity determining residues of the motif are underlined and in bold

The degeneracy of motif-binding domain specificity provides substantial flexibility for a motif-containing peptide to encode a range of binding attributes. Consequently, evolution can adjust the affinity, specificity and selectivity of each domain-motif interaction in the network [10, 47–49]. For example, the affinities of PxIxIT docking motifs for calcineurin can range over two orders of magnitude [50]; artificially increasing the affinity of the PxIxIT motif in the calcineurin-activated transcriptional regulator CRZ1 (Crz1) results in constitutive dephosphorylation, transcriptional hyperactivity, and disruption of other calcineurin-dependent events [51]. This suggests that motif instances in the calcineurin substrate network may have been tuned to optimally regulate substrate modification state. Similarly, the affinity of a PxxP motif in the MAP kinase kinase PBS2 (Pbs2) for its target SRC Homology 3 (SH3) domain in yeast high osmolarity signaling protein SHO1 (Sho1) correlates linearly with the biological output of the high osmolarity glycerol pathway, suggesting that evolution tuned this response by optimising the strength of the interaction [52]. The same motif was shown to bind exclusively to the Sho1 SH3 domain in yeast, but to multiple non-yeast SH3 domains, indicating that evolution has tweaked the motif-domain interface to reduce deleterious promiscuous binding to other co-localised SH3 domains in the yeast proteome [53]. A further level of motif tuning occurs through the acquisition of additional, co-operative motifs (Fig. 2d-f) (see Table 1). For example, the addition of a cluster of Cdk1 consensus sites to the flanks of a pre-existing nuclear localisation signal (NLS) adds a novel level of regulation to the nucleocytoplasmic shuttling of DNA replication licensing factor MCM3 (Mcm3) in yeast [54]. Similar switching mechanisms involving co-operative and competitive use of motifs have evolved on numerous occasions [12, 27, 55, 56]. Remarkably, complete multi-motif interfaces can be acquired relatively rapidly on an evolutionary timescale, for example, the sequential recruitment of motif-binding partners to the multi-motif interfaces regulating the degradation of yeast Cell division control protein 6 (Cdc6) [57] and N-acetyltransferase ECO1 (Eco1) [58].

## What are the evolutionary forces that drive specific motif evolution?

### Ex nihilo motif birth

**Fig. 3:**
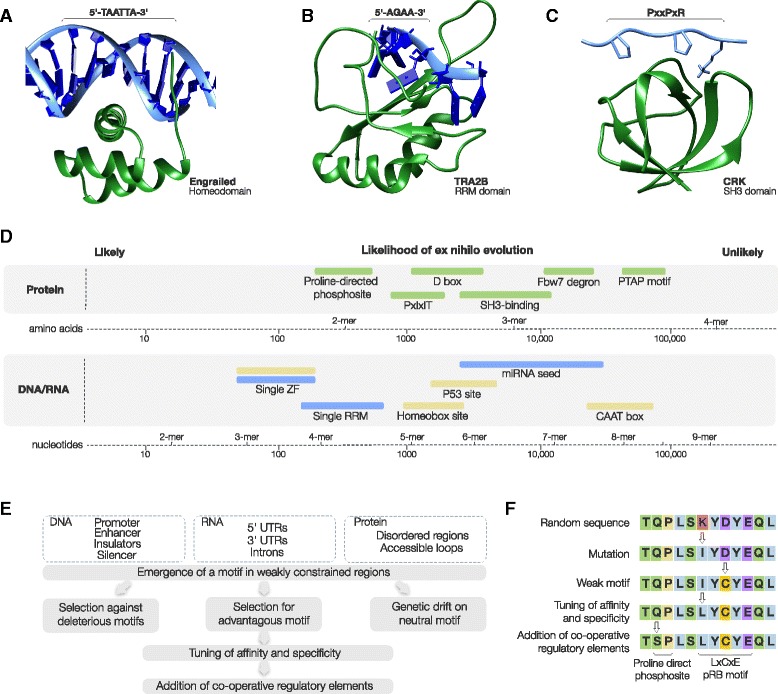
The relationship between compact degenerate motifs, occurrence likelihoods and *ex nihilo* evolution. **a** The homeodomain of *Drosophila* Segmentation polarity homeobox protein engrailed (en) bound to a TAATTA subsite [119]. **b** The RRM of Transformer-2 protein homolog beta (TRA2B) bound to an AGAA exonic splicing enhancer (ESE) motif [120]. **c** The SH3 domain of Adapter molecule crk (CRK) bound to a PxxP motif from Rap guanine nucleotide exchange factor 1 (RAPGEF1) [121]. **d** The number of nucleotides or residues expected between instances of a motif occurring by chance in a sequence. A non-degenerate x-mer nucleotide motif instance would be expected to occur once every 4^x^ nucleotides (e.g. a 6-mer every 4^6^ or 4,096 nucleotides) and an non-degenerate x-mer protein motif would be expected to occur once every 20^x^ amino acids (e.g. a 3-mer peptide motif every 20^3^ or 8000 amino acids). The disparity in the length of the regions that contain these motifs (DNA, (pre-)mRNA and proteins) means that the number of random instances will vary by several fold across the three classes of biomolecule. Ranges are illustrative and are therefore approximate, based on over predictive consensuses (see motifs below) and use equal nucleotide (1/4) and amino acid (1/20) frequencies. Protein SLiMs: proline-directed phosphosite ([ST]P) [29]; D box degron (RxxLxx[ILMVK]) [69]; PxIxIT Calcineurin docking motif (Px[IVLF]x[IVLF][TSHEDQNKR]) [27]; SH3 domain-binding motif (PxxPx[KR]) [32]; PTAP late domain motif (P[TS]AP) [122]; and Fbw7 SCF degron([ILMVP]TPxx[ST]) [123]. RNA motif: A single RRM binding site (4 nucleotides) [124]; a single Zinc Finger recognition site (3 nucleotides) [125]; and an miRNA seed regions (6–8 nucleotides) [126]. DNA motifs: a single Zinc Finger recognition site (3 nucleotides) [127]; Homeobox domain (TAAT[GT][GT]) [128]; CAAT box ([TC]GATTGG[TC][TC][AG]) [129]; and P53 regulatory element (C[AT][AT]GNNNNNNC[AT][AT]G) [130]. **e** Simple model for motif acquisition by DNA, RNA and proteins (see text for details of model). **f** Potential mechanism of *ex nihilo* motif evolution illustrated using a hypothetical LxCxE pRB-binding motif (see text for details of model)

In contrast to protein domain evolution - which is driven by duplication, recombination and divergence [59, 60] - we still lack a clear understanding of the mechanisms driving SLiM evolution. To understand the mechanism of *ex nihilo* motif birth we must consider two major observations about SLiMs: (i) like the analogous motifs in the regulatory regions of DNA and (pre-)mRNA, they are compact and degenerate [13] (Fig. 3a-c); and (ii) they usually occur in rapidly evolving intrinsically disordered regions [13, 61, 62]. The majority of SLiM-binding domains have weak specificity, because they contact a core motif of only three to four residues, and often tolerate amino acids in these positions that have similar physicochemical properties [13]. Similarly, there are few restrictions on the amino acids that flank the motif, although these residues can indirectly modulate the physical, chemical or structural compatibility of the peptide with the target domain (Fig. 1d) [10, 13, 14, 63]. Consequently, the motif core is necessary but not sufficient for binding and many bone-fide motif instances fail to conform to the consensus sequence. Given these limited specificity and affinity determinants of the motif, they are expected to occur frequently by chance (Fig. 3d) [13], and a proteome will contain many peptides that are complementary to the motif-binding pocket (though many of these sequences will never meet their binding partner in the cell due to temporal and spatial restrictions [64]). Because much of the intrinsically disordered regions of a proteome are apparently under weak selective constraints and are rapidly changing at the sequence level [61], mutations, insertions and deletions in these regions facilitate the rapid sampling of sequence space. Taken together, the simplicity of the motif and the rapid evolution of disordered regions drive a system where peptides complementary to the binding pocket of a given SLiM-binding domain are rapidly being created, by *ex nihilo* motif birth, and destroyed. This ever-changing set of motifs may represent a dynamic evolutionary reservoir of new protein-protein interactions that fuel selectable phenotypic diversity.

### Motif fixation

Motif birth occurs as a single mutation in a single allele in a single member of a species. When studying motifs, we generally consider a motif present in a fixed allele (i.e. it is present in all members of the population – SLiM-containing alleles may also be subject to balancing selection though no examples are known). On a population level, the steps from the *ex nihilo* birth of a motif to fixation or loss can follow several paths (Fig. 3e). The likelihood of motif fixation or loss will be dependent on the phenotype of the motif and the effective population size [65]. For clarity three basic groupings can be used to describe a continuum of motif phenotypes: beneficial motifs are those that have an adaptive phenotype; neutral motifs are those that do not have any selectable positive or negative phenotype; and deleterious motifs are those that have a selectable negative phenotype. As a general model, alleles with beneficial motifs will be under positive selection and will become fixed in the population; those with neutral motifs can become fixed or lost by genetic drift; and those with deleterious motifs will be lost by negative selection. However, due to stochasticity in the evolutionary process, exceptions will occur. For example, beneficial motifs can be lost by genetic drift before they reach appreciable frequencies and deleterious motifs can become fixed in small populations. Once a motif has become fixed, negative (or purifying) selection will retain beneficial motifs, and subsequent mutations that become fixed by genetic drift will tend to remove neutral motifs over time. Substitutions that deleteriously affect the affinity, specificity and selectivity of a beneficial motif will generally be under negative selection and will fail to spread through the population. Conversely, those that result in a superior phenotype will be under positive selection and can become fixed. The interplay of this positive and negative selection might give directionality to the evolution of a motif and could in effect act as a ratchet to optimise the motif’s binding attributes (Fig. 3f).

### Motif optimization in a network

Multiple motif-containing proteins are often competing for a finite pool of a given motif-binding pocket-containing protein. The optimisation of each motif must thus be considered in the context of the whole interaction network: to balance competition between motif-containing proteins and define the proportion of each motif-containing protein that occupies a given motif-binding pocket. These systems must consider the timing/strength of expression of the motif-containing and motif-binding partners and, as many motifs function in multiprotein complexes and cannot sustain interactions without co-operativity, changes in expression of scaffolding molecules. Such a model would require co-evolution of the network to tune the attributes of each interface in reaction to changes to the network. These network changes can include: an increase or decrease in the abundance of a component of the network; the gain or loss of a motif; mutations that alter the affinity, specificity and selectivity of a motif; or the addition of intramolecular co-operativity between motifs that can increase the avidity of an interaction, increase the specificity of an interaction, or add regulatory constraints that act as conditional modulators of an interaction [51, 66]. Many inhibitors of motif-mediated systems, both endogenous and pathogenic, take advantage of the delicate balance of these systems by utilising high affinity motifs, or high avidity co-operative multi-motif interfaces, to titrate the available motif-binding proteins [46, 67–69]. A related question is whether the cumulative effect of all presumably individually neutral motifs on the network level can have an appreciable phenotype by titrating the motif-binding partner away from motif-containing proteins. A consequence of this would be that there exists an upper limit to the number of instances of a motif in a proteome. It is evident that large numbers of motif instances for a single motif-binding partner are possible, for example, NLS motifs are present in hundreds of proteins yet they function without issue [70]. However, it has also been shown that motif–containing peptides in high concentrations can act as potent inhibitors [71]. Similar inhibitory effects have been observed for motifs with artificially increased affinities [51]. Several motif networks have been shown to recruit targets with a hierarchy driven by the intrinsic affinity for their motif-containing binding partner. In some cases, these networks regulate recruitment using competitive mechanisms facilitated by limiting amounts of the motif-binding domains [72]. So can evolutionarily neutral motif instances in sufficiently high quantities or with sufficiently high affinities act as inhibitors? Or would the set of novel untuned, and therefore possibly lower affinity, motifs be outcompeted by the key biological targets? This is currently unclear. However, the upper limit of instances of a functionally important motif is likely correlated with the abundance of the motif-binding protein and the abundance and relative affinities of the motif-containing proteins. An important consideration is that motif-binding domains instances, in excess, can significantly bind a pool of weaker motifs beyond their normal targets [66]. Perhaps the expansion of a motif network is the result of an increase in the abundance of the motif-binding partner, and thus an expansion of the number of recruited motif-containing proteins, followed by a wave of selection. These concepts illustrate that when considering the evolutionary forces of mutations in motifs it is important to consider both protein autonomous effects (i.e., changes in the regulation of that protein) and effects due to modulation of the larger protein interaction network.

## What is the mechanism of motif-binding pocket evolution?

**Fig. 4:**
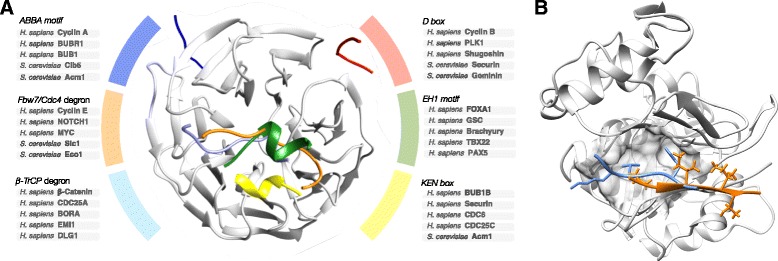
Examples of motif-binding pocket evolution. **a** Representative selection of motif-binding pockets in the WD40 repeat fold demonstrating the simplicity of motif-binding pocket birth. Each pocket has evolved independently and subsequently multiple proteins (representative examples listed) have acquired the motifs necessary to recruit the various WD40 repeat containing proteins. The figure includes: an ABBA motif (*dark blue* – consensus [ILV][FHY]x[DE]), a D box degron motif (*red* – consensus RxxLxx[ILVK]) and a KEN box degron motif (*yellow* – consensus KEN) from APC/C-CDH1 modulator 1 (Acm1) bound to the WD40 domain of the APC/C activator protein CDH1 (Cdh1) [69]; an Fbw7 degron motif (orange – consensus _p_TPxx_p_S) from Cyclin E bound to the WD40 domain of the F-box/WD repeat-containing protein 7 (FBW7) [123]; a β-TrCP1 degron motif (*light blue* – consensus D_p_SGxx_p_S) from β-Catenin bound to the WD40 domain of the F-box/WD repeat-containing protein 1A (BTRC) [131]; and an EH1 motif (*green* – consensus [FHY]x[IVM]xx[ILM][ILMV]) bound to the WD40 domain of the Transducin-like enhancer protein 1 (TLE) [132]. See the ELM resource for more details and examples [9]. **b** Example of specificity divergence after motif–binding domain duplication. A homologous pocket on the protein phosphatase 1 (PP1) and calcineurin holoenzymes bind RVxF and PxIxIT motifs respectively. The structure shows the canonical PP1 binding sequence RVxF motif (*light blu*e) of myosin phosphatase targeting subunit (MYPT1) bound to PP1 (*grey*). The PxIxIT of African swine fever virus A238L (A238L) (*orange*) is superimposed showing the shared but diverged binding pocket [115]. The valine and phenylalanine of the RVxF motif sit in the hydrophobic P1 and P3 regions occupied by the proline and first isoleucine of the PxIxIT binding pocket (see Fig. 1d) but the additional specificity/affinity determinants of the two motifs utilise different surfaces of the domain and do not overlap [50, 133]

Where do motif-binding pockets come from in the first place? A potential model of motif-binding pocket gain is that coevolution of the original binding partner(s) and the binding pocket optimises a surface for motif binding and, subsequently, additional peptides utilise the pocket to recruit the protein. The outcome of the reuse of the binding pocket by multiple distinct binding partners and the required complementarity between binding peptide and the binding pocket results in the repeated patterns that we refer to as motifs. Motif pocket birth has been observed for many domain families (e.g. the RNA recognition motif domain (RRM) and the WD40 repeat) where a family member acquires a novel motif-binding pocket (Fig. 4a) [73, 74]. A recent study presented structural and functional evidence for a derived docking-motif binding-pocket in the highly conserved kinase domain of yeast serine/threonine-protein kinase CBK1 (Cbk1) [75]. In this case, after evolution of the binding pocket, docking motifs appear to have arisen ex nihilo in disordered regions of proteins that were already Cbk1 substrates, and were subsequently preserved over evolution. Thus, fungal Cbk1 offers a rare example where the evolution of an entire SLiM-pocket interaction network has been traced. Once established, a SLiM binding pocket is generally conserved over large evolutionary distances as the motif partners constrain the pocket (unless the domain duplicates). For example, the NLS of human Myc proto-oncogene protein (MYC) can be recognised by importin subunit alpha (Srp1) of the yeast nuclear import machinery [76]. Conversely, co-evolution can also maintain critical binding interactions as the peptide binding domain specificity changes. This process of domain-motif co-evolution, where the motif recognised by a binding pocket and the binding pocket drift on the sequence level, has been observed in a few cases, such as the PCNA-binding PIP boxes [77] and the APC/C activator protein CDC20-binding ABBA motif [40, 78] in the fungal lineage.

Some motif-binding domains are members of large domain families. Members of most of these motif-binding domain families, while utilising the same binding pocket, have diverged specificities to recognise distinct, often overlapping, sets of peptides (Fig. 4b) [10, 75, 79, 80]. For example, the optimal specificities of kinases [81–83] and SRC Homology 2 (SH2) domains [84, 85] have diversified during family expansions. The specificity of a motif-binding pocket is dependent on its physicochemical properties. Evolutionary refinement of the domain surface post-duplication can modulate these physicochemical properties and thus the binding preferences of the motif-binding domain. For example, dependent on the biological requirement, amino acid changes in the binding surfaces can shift the binding preferences to allow a given peptide bind to one of the duplicated domains but not the other, or less drastically, bind with different affinities to each domain. Both mechanisms result in diverged specificities for the novel binding domains and over time the specificity of the domains can drift extensively. When overlapping specificity with homologous, or non-homologous, co-localised domains results in deleterious motif-binding events the specificities of motif-binding pockets will evolve to reduce this overlap [48, 53, 83, 86]. For example, mitotic kinases have been observed to target the correct substrates by a combination of substrate co-localisation and kinase specificity. The specificity of several of these kinases have evolved to specifically disfavour the motifs of other co-localised mitotic kinases [48, 87].

## When did extensive motif use evolve?

The diversity of the physicochemical properties of SLiMs is remarkable and it seems the only limit on the evolution of novel and distinct motif classes may be that the reuse of currently available motif-binding domains and the subtle tweaking of their specificity is sufficient in most cases. Nevertheless, when required evolution can and does innovate, however, often that innovation can use similar building blocks [73, 74]. A significant portion of the higher eukaryotic motif space (the set of motifs with the ability to specifically bind a SLiM-binding pocket) is now utilised by SLiM-binding domain families [9, 88–90]. However, the exact timing of the explosion of SLiM use is unknown. Archaea and Bacteria use motifs, for example the sliding clamp binding motif [91] and several motifs in the degradosome protein Rnase E [92], but not to the same extent as Eukaryotes. Interestingly, this is reflected in the relative levels of intrinsic disorder in these domains of life [93], however, this relationship between the expansion of motif use and intrinsic disorder is still unstudied. The sporadic evolution of novel motif-binding pockets in domains that previously had no SLiM-binding ability has contributed to the diversity of SLiM-binding [73, 74]. However, much of the growth of motif space coincided with the expansion of the large canonical motif-binding domain (e.g. SH3) and motif-modifying domain (e.g. kinase) families in Eukaryotes (Table 2). An expansion that mirrors that of the canonical DNA and RNA motif-binding families. A common theme for these families is the duplication of a domain followed by the divergence of the specificity of the resulting domains. This has resulted in a complex landscape of specificities for many of the large motif-binding families in higher Eukaryotes [10]. Because most of these domain families were present in distantly related Eukaryotes, and many rapidly expanded thereafter, the general consensus is that extensive motif usage evolved very early in eukaryotic evolution [85, 94] and the diversity of motif types has continued to expand with the diversification of the motif-binding and motif-modifying domains [83, 85, 95]. Expansions of a given motif-binding domain family may also be specific to certain lineages [95, 96]. For example, the motif-binding SH2 and SH3 domains, key metazoan signalling components, are rare in plant proteomes [95].

**Table 2:** Table of several classical SLiM-binding domain families, and representative DNA and RNA motif-binding domain families^a^

Domain type	Domain	Ath	Ddi	Sce	Dme	Hsa
*SLiM-binding*	SH3 domain	5	29	23	59	204
	PDZ domain	17	1	2	67	145
	SH2 domain	2	13	0	32	110
	WW domain	9	5	6	21	41
	Kinase domain	1066	309	132	289	523
*DNA-binding*	C2H2/C2HC zinc finger	22	6	34	197	659
*RNA-binding*	RRM domain	268	96	58	137	265

^a^The number of instances of each family in *Arabidopsis thaliana* (Ath), *Dictyostelium discoideum* (Ddi), *Saccharomyces cerevisiae* (Sce), *Drosophila melanogaster* (Dme), *Homo sapiens* (Hsa). Data from *Vogel et al.* [95]

## Do common principles of regulatory evolution unite motifs in DNA, RNA and Protein?

Many parallels have been observed for motif use at the transcriptional, post-transcriptional and post-translational level. For example, specification of responses through the co-operative action of multiple motif recruited regulators is a theme at all levels of regulation (transcription: [97], splicing: [98], miRNA [99], signalling [11]). Much like combinations of SLiMs in disordered regions that lead to combinatorial post-translational regulatory switches [55], enhancers integrate complex transcriptional circuitry to individual genes [97]. Like the regulatory regions of DNA and (pre-)mRNA, disordered regions containing multiple SLiMs are key foci where the gain and loss of motifs can lead to complex changes in cell regulation and physiology [38, 68]. Another example is the analogy of SLiM-binding pocket and SLiM co-evolution with DNA-binding domain - DNA regulatory element co-evolution. Because of the predicted pleiotropy of DNA-binding domain specificity changes, it was argued that such changes (in trans) should be comparatively rare relative to changes in the modular DNA binding sites (in cis [18]). Nevertheless, several examples of such changes and the corresponding co-evolution of DNA binding sites were subsequently identified (e.g., [100]). Once again, examples of pocket-SLiM co-evolution exist [40, 77, 78]. Finally, recent genome-scale chromatin immunoprecipitation and DNase hypersensitivity mapping experiments have indicated that DNA-protein interactions evolve rapidly between species. These results suggest that many DNA motif - protein interactions in complex genomes are not preserved over evolution while a small subset of functional binding sites is preserved near key target genes [101]. This is analogous to the evolutionary reservoir model described above, where most SLiMs are evolutionarily transient, and a few core SLiMs are preserved by natural selection. The rapid evolutionary turnover of a large fraction of regulatory interactions is consistent with a model where most of the changes are nearly neutral with respect to selection [65, 102] (although we note that extensive lineage-specific selection could also produce similar patterns [103]). If the mostly neutral model is correct, only a small fraction of the evolutionary reservoir created by non-adaptive processes will be preserved by natural selection. Due to the size and complexity of eukaryotic genomes and proteomes and the short, degenerate nature of motifs, the rate of *ex nihilo* motif gain may be rapid enough that a large number of neutral regulatory interactions are present at all levels (DNA, RNA and proteins).

## Conclusion

Every motif will be subjected to unique evolutionary pressures and novel motifs will fall along a phenotypic continuum rather than a neatly classifiable trinity of positive, neutral or negative phenotypes. Nevertheless, we have described a general model for the mechanism of motif evolution where the dynamic equilibrium of motifs being rapidly created *ex nihilo* in disordered regions and then destroyed by mutations provides a reservoir of functional diversity in protein interaction networks. We believe this diversity represents a key raw material exploited by evolution as it elaborates the complexity of the cell. This advocates a model of protein evolution resulting from both domain duplication and *ex nihilo* motif evolution.

The expansion of motif-binding domains linking compact and degenerate peptides to important functions greatly increased the information processing potential of the cell by simplifying access to regulatory pathways and cell state information. This expansion of functional motif space has allowed mutations, insertions and deletions to act as a powerful mechanism to add novel functional modules to a protein. Such a simple evolutionary mechanism to create selectable phenotypic diversity appears to have been advantageous to many organisms as it was extensively expanded and exploited resulting in an explosion in network connectivity and an increase in the regulatory complexity of the cell. The large functional motif space also increased the evolvability of these organisms by offering huge potential future adaptive evolution. Thus, it is tempting to assume that increasing motif usage is beneficial to complex organisms. However, as the Noonan-like syndrome motif “knock in” example shows, on an individual level, the deleterious effect of motif birth can be severe. The relative likelihood of motif gain and loss is still unknown, however, it is possible that if the effective population size becomes small for complex organisms, and interactions may appear *ex nihilo* in disordered regions at a high enough rate, natural selection might simply not be strong enough to purge them. [65, 104].

Many basic questions remain regarding the extent of motif use. How many motifs specifically bind each motif-binding pocket? How many of these motif-binding events are biologically important? How many are “evolutionary noise” [65]? These unknowns complicate our quest to understand motif evolution and consequently numerous unanswered evolutionary questions also exist. How often do motifs arise *ex nihilo*? What proportion of these novel motifs are advantageous, deleterious and neutral? What is the cumulative cost of multiple neutral motifs? If the acquisition of a given motif class is advantageous to a particular protein will it eventually acquire it? How does evolution optimise the binding attributes of a motif? How do co-operative sets of motifs evolve (Does the presence of a motif increase the likelihood of the acquisition of a co-operative motif)? Further experimental and theoretical exploration is needed to answer these questions. This will be confounded by experimental limitations (perhaps “biologically irrelevant” motifs haven’t been tested under the correct lab conditions) and the weak phenotypes, redundancy and co-operativity of many motifs. This remains a key area of research and will require numerous experimental and analytical advances. A key step will be the creation of unbiased, proteome-wide approaches to identify SLiMs, such as proteomic phage display [105, 106]. Although the experimental and analytical techniques will be specific to SLiMs, in light of the parallels between regulatory motifs in all the major macromolecules, we suggest that studies aimed at understanding the mechanisms of SLiM evolution should consider their evolutionarily analogous motifs in the regulatory regions of DNA and (pre-)mRNA. Ultimately, our understanding of cell regulation could benefit greatly through the use of shared concepts and models for motif evolution at the transcriptional, post-transcriptional and post-translational level (e.g., [35, 65, 107]).
